# Optic Nerve Head Parameters in a Turkish Population Over Forty Years of Age

**DOI:** 10.4274/tjo.89847

**Published:** 2016-08-15

**Authors:** Leyla Niyaz, Nilgün Yıldırım, Ahmet Musmul

**Affiliations:** 1 Ondokuz Mayıs University Faculty of Medicine, Department of Ophthalmology, Samsun, Turkey; 2 Osmangazi University Faculty of Medicine, Department of Ophthalmology, Eskişehir, Turkey; 3 Osmangazi University Faculty of Medicine, Department of Biostatistics and Medical Informatics, Eskişehir, Turkey

**Keywords:** Optic disc, optic cup, disc area, cup area

## Abstract

**Objectives:**

To evaluate the optic disc area and cup area in a normal population over 40 years of age.

**Materials and Methods:**

This prospective study was performed in Eskişehir. Fundus photographs were obtained using a nonmydriatic fundus camera. Planimetric measurements of the optic disc and cup area were performed with VK-2 digital imaging software. Optic nerve parameters were then compared between sex and age groups.

**Results:**

A total of 3,038 subjects were evaluated. Mean age was 56.6±10.4 years (range 40-91 years). The median disc area of the subjects was 2.87 (2.53-3.23) mm^2^ in the right eyes and 2.89 (2.55-3.25) mm^2^ in the left eyes (p<0.001). The median cup area of the subjects was 0.46 mm^2^ (0.33-0.64 mm^2^) in the right eyes and 0.44 mm^2^ (0.33-0.61 mm^2^) in the left eyes (p<0.001). The differences in disc and cup area between male and female subjects were not statistically significant (p>0.05).

**Conclusion:**

We report the normal distribution of disc area and cup area measurements and their association with age and sex.

## INTRODUCTION

Morphological changes in the optic nerve head have diagnostic value for many systemic and ocular disorders. Disc size and cup-to-disc ratio are important parameters in the assessment of disc damage in glaucoma.^1,2^ The normal disc size varies between 0.8 and 6.0 mm^2^ and can depend on underlying pathologies such as myopia, hyperopia, morning glory anomaly, optic disc drusen and papilledema. There is also a pronounced interindividual variability of disc size due to ethnic variation, equipment used and physicians making the evaluation.^3,4,5,6,7^

Since data on disc parameters in the Turkish population are scarce, the present investigation was conducted to examine optic disc and cup areas in Turkish people over 40 years of age.

## MATERIALS AND METHODS

This study was a population-based study conducted in Eskişehir. We evaluated both eyes of subjects over age 40 who were reached through registrations in the Health Centers in one township (Kaymaz) and three districts (Esentepe, Şirintepe, Osmangazi) of Eskişehir. After obtaining informed consent, a questionnaire was performed by a professional technician who had more than five years of experience in ophthalmology. Ocular and systemic pathologies as well as demographic factors such as age and sex were noted. Intraocular pressure was measured by Tono-pen (Medtronic Ophthalmics, Jacksonville, Florida, USA) and central corneal thickness by Pacline pachymeter (Opticon 2000 S.p.A., Rome, Italy). Fundus photographs were then taken by a KOWA (Kowa, Nagoya, Japan) nonmydriatic fundus camera. Pupils were not dilated. A total of 3,038 subjects with sufficient media clarity to permit good quality fundus photographs underwent photography.

**Figure 1:**
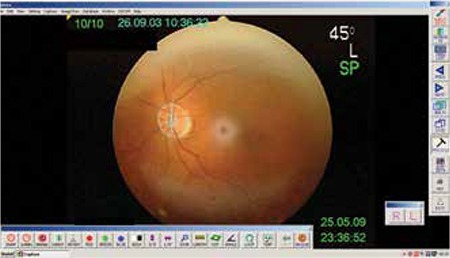
Drawing of the optic nerve head with peripapillary atrophy

The fundus images of 3,038 cases were assessed by planimetric measurements using 45º photographs. Borders of the discs and cups were manually marked at 12 points by a researcher and a line was automatically generated by the VK-2 digital imaging software (Figure 1). The cup was outlined as the central depressed area and the site of bending of the vessels was accepted as the true margin. The border of the disc was marked at the inner side of peripapillary scleral ring. If present, parapapillary alpha atrophy with irregularities of the retinal pigment epithelium and beta atrophy with absent pigment epithelium and visible choroidal vessels were left outside the drawn borders. In cases with tilted discs, the scleral ring or edge of the retinal pigment epithelial/choriocapillaris layer were accepted as the disc margin. Nonmydriatic fundus camera parameters included disc area, cup area and cup/disc area ratio.

The study population was divided into four groups according to age: 40-49 years; 50-59 years; 60-69 years; and over 70 years. Both eyes of all cases were evaluated. Eyes with glaucoma and eyes without clear fundus images that prevented reliable planimetric assessment were excluded from the study.

Descriptive statistics were used to describe each variable. The descriptive statistisics were demonstrated with n (sample size), mean and standard deviation for continuous variables, and n (sample size), median and 25^th^ (Q1) and 75^th^ (Q3) percentiles for non-normally distributed variables. Kolmogorov-Smirnov and Shapiro-Wilk tests of normality were used to check the normality of variable distributions. Independent variables that did not show normal distribution were compared using Kruskal-Wallis and Mann-Whitney U tests. Wilcoxon Signed Ranks test was used for the dependent non-normally distributed variables. A value of p<0.05 was accepted as significant. SPSS 21.0 program was used for statistical analysis.

## RESULTS

A total of 2,862 right and 2,856 left eyes of 3,038 subjects were evaluated. There were 2,241 female and 797 male subjects in the study. Overall mean age was 56.6±10.4 years (range 40-91 years). The mean age of female subjects was 55.6±10.1 years and the mean age of the male subjects was 59.2±10.5 years. Mean intraocular pressure in right and left eyes was 16.2 mmHg and 16.1 mmHg, respectively. The frequency of tilted disc, alpha and beta atrophies was 0.94%, 22% and 7.45%, respectively.

### Optic Disc

The median disc area of the subjects was 2.87 (2.53-3.23) mm^2^ in the right eyes and 2.89 (2.55-3.25) mm^2^ in the left eyes (p<0.001). Median disc areas in females were 2.87 (2.57-3.32) mm^2^ and 2.90 (2.60-3.24) mm^2^ in right and left eyes respectively. Median disc areas in males were 2.87 (2.53-3.23) mm^2^ and 2.89 (2.55-3.25) mm^2^ in right and left eyes respectively. The difference between male and female subjects was not significant (p=0.25 and p=0.19 for right and left eyes, respectively).

Disc areas according to the age groups are given in Table 1. There was no significant difference between the groups (p>0.05). The difference between right and left eyes was significant in the 50-59 and 60-69 age groups.

Disc areas were observed to be larger in the younger and older age groups (p>0.05), but the difference was only found to be significant between 60-69 vs 70+ groups in female subjects (p=0.029) (Table 2).

#### Optic Cup

Optic cup area ranged from 0.33 to 0.64 mm^2^ (median 0.46 mm^2^) in the right eyes and from 0.33 to 0.61 mm^2^ (median 0.44 mm^2^) in the left eyes (p<0.001). Median cup areas in females were 0.45 (0.34-0.63) mm^2^ and 0.44 (0.33-0.61) mm^2^ in right and left eyes respectively. Median cup areas in males were 0.46 (0.32-0.63) mm^2^ and 0.45 (0.33-0.61) mm^2^ in right and left eyes respectively. The difference between male and female subjects was not significant (p=0.49 and p=0.73 for right and left eyes respectively).

There was a significant correlation between disc area and cup area in the right and left eyes (p<0.001).

## DISCUSSION

Optic nerve head assessment is important in many ocular and systemic diseases, especially in glaucoma, which is a well known preventable cause of blindness. To detect early pathological changes in the optic nerve head, one must know the normal distribution of its parameters. There are many studies in this area, but almost none in Turkish population.^8,9^

In this study we evaluated optic disc and cup area in a Turkish population. We used a nonmydriatic fundus camera to evaluate the optic nerve head of the subjects. It is a noninvasive, noncontact imaging technique that does not require pupillary mydriasis. Fundus cameras have become increasingly automated and they include computer-assisted digital analysis of the optic nerve head. Computer-assisted techniques have improved intra-observer reproducibility and inter-observer consistency.^10^

We found the mean optic disc area to be 2.88 mm2 (1.2-5.28 mm^2^). In the Beijing Eye study performed on the Chinese population, mean disc area was 2.65 mm^2^ (1.03-7.75 mm^2^). In the Indian population it was 3.37 mm2 (2.04-4.7 mm^2^). In all of these studies, planimetric measurements were used.^11,12^ Marsh et al.^7^ showed that the mean optic disc area was significantly smaller in white and Hispanic subjects than in African-Americans. Durukan et al.^13^ found the mean disc area to be 2.12 mm^2^ in a normal Turkish population, but the measurements were done using a confocal scanning laser ophthalmoscope (HRT II), and this may explain the difference from our study. Thomas et al.^14^ compared HRT and planimetric findings and concluded that disc area is measured smaller by HRT than by planimetry (2.24 vs 2.58 mm^2^). It is well known that the technique and equipment used have an important influence on measurement results.

In our study, the mean disc area was 2.89±0.45 mm^2^ in male subjects and 2.88±0.46 mm^2^ in female subjects. Although female subjects had smaller discs, the difference was not significant (p>0.05). Varma et al.^15^ studied 3,387 normal American subjects and concluded that males had larger discs than females in both the white and black populations. In contrast, there are other studies which do not support this evidence.^12,16^

We did not find a significant difference between age groups in terms of disc area or cup area. The only significant difference was observed for disc area between the 60-69 and 70+ age groups in female subjects, which might be coincidental. Durukan et al.^13^ found a significant difference between subjects over and under 60 years of age, with larger disc areas in subjects over 60 years. In a postmortem study performed by Quigley et al.,^8^ no relation was found between age and disc size. The same results were found in the Beijing Eye Study and Baltimore Study.15,17 This may show that the effect of age on disc size may vary among different nations.

In our study, mean cup area was measured as 0.45 mm^2^ and we did not find any significant differences in the measurements between sexes. Similar results were observed by Durukan et al.^13^ In a study by Xu et al.,^9^ the mean cup area of a Chinese population was larger than that of our population, which may be attributable to ethnic differences.

There are some limitations of this study. The evaluation technique requires a high degree of subjective judgment and slight differences in settings used may cause variation between centers. Keratometric readings of patients’ corneas or measurements of the axial lengths were not available; therefore, an individual correction for the magnification of the optics of the eye was not possible.

## CONCLUSION

We report the normal distribution of disc area and cup area measurements in the Turkish population. Disc area and cup area did not vary significantly between male and female subjects or between different age groups.

### Ethics

Ethics Committee Approval: Approval number 2008/263, Informed Consent: It was taken.

Peer-review: Externally and internally peer-reviewed.

## Figures and Tables

**Table 1:**
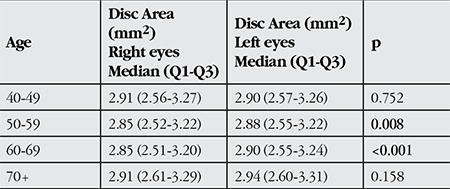
Mean disc areas in the right and left eyes according to the age groups

**Table 2:**
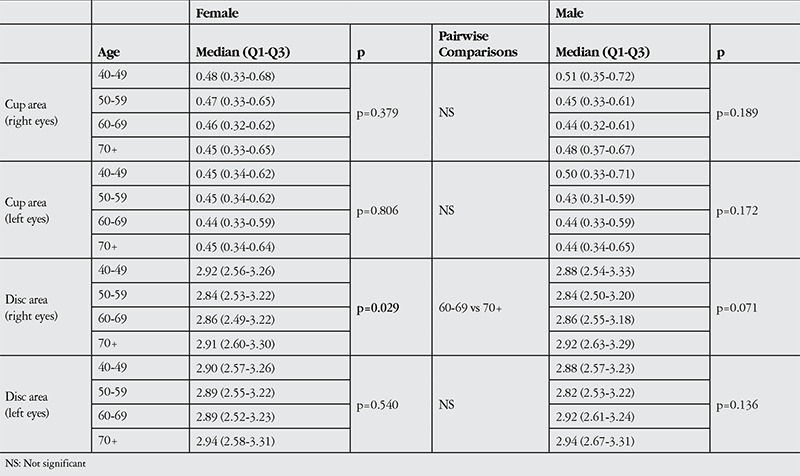
Disc parameters in males and females according to the age groups
